# Targeting *Pseudomonas aeruginosa* in the Sputum of Primary Ciliary Dyskinesia Patients with a Combinatorial Strategy Having Antibacterial and Anti-Virulence Potential

**DOI:** 10.3390/ijms21010069

**Published:** 2019-12-20

**Authors:** Giuseppantonio Maisetta, Lucia Grassi, Semih Esin, Esingül Kaya, Andrea Morelli, Dario Puppi, Martina Piras, Federica Chiellini, Massimo Pifferi, Giovanna Batoni

**Affiliations:** 1 Department of Translational Research and New Technologies in Medicine and Surgery, University of Pisa, 56123 Pisa, Italy; lucia.grassi@med.unipi.it (L.G.); semih.esin@med.unipi.it (S.E.); esingulkaya@gmail.com (E.K.); giovanna.batoni@med.unipi.it (G.B.); 2 Department of Chemistry and Industrial Chemistry, University of Pisa, 56124 Pisa, Italy; a.morelli@dcci.unipi.it (A.M.); d.puppi@dcci.unipi.it (D.P.); federica.chiellini@unipi.it (F.C.); 3 Section of Pneumology and Allergology, Unit of Pediatrics, Pisa University Hospital, 56126 Pisa, Italy; martinaprs@gmail.com (M.P.); m.pifferi@med.unipi.it (M.P.)

**Keywords:** antimicrobial peptide, EDTA, *Pseudomonas aeruginosa*, primary ciliary dyskinesia, virulence factor, anti-virulence, sputum, chronic infection

## Abstract

In primary ciliary dyskinesia (PCD) patients, *Pseudomonas aeruginosa* is a major opportunistic pathogen, frequently involved in chronic infections of the lower airways. Infections by this bacterial species correlates with a worsening clinical prognosis and recalcitrance to currently available therapeutics. The antimicrobial peptide, lin-SB056-1, in combination with the cation chelator ethylenediaminetetraacetic acid (EDTA), was previously demonstrated to be bactericidal against *P. aeruginosa* in an artificial sputum medium. The purpose of this study was to validate the anti-*P. aeruginosa* activity of such a combination in PCD sputum and to evaluate the in vitro anti-virulence effects of EDTA. In combination with EDTA, lin-SB056-1 was able to significantly reduce the load of endogenous *P. aeruginosa* ex vivo in the sputum of PCD patients. In addition, EDTA markedly reduced the production of relevant bacterial virulence factors (e.g., pyocyanin, proteases, LasA) in vitro by two representative mucoid strains of *P. aeruginosa* isolated from the sputum of PCD patients. These results indicate that the lin-SB056-1/EDTA combination may exert a dual antimicrobial and anti-virulence action against *P. aeruginosa*, suggesting a therapeutic potential against chronic airway infections sustained by this bacterium.

## 1. Introduction

Primary ciliary dyskinesia (PCD) is an autosomal recessive disorder characterized by abnormal ciliary ultrastructure and function leading to impaired mucociliary clearance and recurrent respiratory infections [1]. Although *Haemophilus influenzae* is the pathogen most commonly isolated from patients with PCD until adolescence/early adulthood, in adult PCD patients, *P. aeruginosa* plays a major role, especially after the age of 30 [1]. Accordingly, a negative correlation between the abundance of *P. aeruginosa* in the airways of these patients and lung function has been reported [2,3]. The pathogenesis of *P. aeruginosa* infection is at least partially attributable to its ability to synthesize and secrete a number of virulence factors (e.g., pyoverdine, pyocyanin, proteases) and to form biofilms, in which bacterial cells are embedded in an alginate extracellular matrix [4]. Despite intensive antibiotic therapy, once the patients are stably colonized by *P. aeruginosa*, the eradication of the bacterium is rarely achieved [1,5]. Therefore, there is a critical need for novel antimicrobial drugs that can effectively lower *P. aeruginosa* load in the challenging environment of PCD lung.

Over the last decades, antimicrobial peptides (AMPs) have been intensively investigated as potential antibiotics against multidrug-resistant bacteria [6]. Most AMPs are cationic molecules with an amphipathic structure that selectively target bacterial membranes via electrostatic forces. In contrast to standard antibiotics, AMPs are generally effective against both quiescent and actively growing bacteria, display rapid killing kinetics, and demonstrate low propensity to select resistant mutants in vitro [7,8]. On the other hand, AMPs may display a reduction in their antibacterial potency in the presence of complex biological fluids such as sputum, plasma, or saliva due to the high concentration of salt found in these fluids and/or the presence of anionic proteins and host or bacterial proteases that may neutralize their activity [9,10].

As observed in cystic fibrosis (CF) lungs, the biofilm mode of growth of bacteria together with the lung mucus viscosity reduces the effectiveness of conventional antibiotic therapy in PCD patients [11]. Thus, the usage of adjuvants has been proposed to improve the diffusion of antimicrobials through the mucus and the biofilm matrix and facilitate the targeting of bacterial cells [12]. Previous studies have shown that the divalent cation chelator ethylenediaminetetraacetic acid (EDTA) can destabilize the biofilm structure by interfering with the ionic attractive forces among the biofilm matrix components [13,14]. EDTA is prescribed in a number of clinical conditions demonstrating high tolerability (up to 2 g once a week, intravenously injected) and efficacy [15]. Recently, we demonstrated that the optimized semi-synthetic antimicrobial peptide lin-SB056-1 in combination with EDTA is able to exert a synergistic bactericidal effect against *P. aeruginosa* in an artificial sputum medium resembling CF sputum [16]. Similarities and differences between CF and PDC sputum have been reported. For instance, while both diseases seem to be associated with a similar degree of airways neutrophilia, the concentration of interleukin-8 in sputum is higher in PCD than in CF patients, while neutrophil elastase activity is lower in PCD compared with CF [17]. In order to evaluate the therapeutic potential of the lin-SB056-1/EDTA combination in PCD, in this study, we evaluated its bactericidal activity ex vivo, against endogenous *P. aeruginosa* in the sputum from PCD patients. Importantly, the ex vivo sputum mimics, with good approximation, the lung environment, as it contains both host and bacterial components, including bronchial mucus contaminated by saliva, serum proteins, inflammatory mediators, desquamated epithelial cells, and pathogenic bacteria, as well as bacteria from the normal flora [18].

Previous reports have demonstrated the involvement of different cations (i.e., calcium, magnesium, and zinc) either in the regulation of gene expression or in the production and processing of virulence factors in *P. aeruginosa* [19,20]. Thus, herein, we also evaluated the ability of EDTA to reduce the production of relevant virulence factors of *P. aeruginosa* (e.g., pyoverdin, pyocyanin, proteases, biofilm production). Overall, the results obtained demonstrated that the lin-SB056-1/EDTA combination is able to significantly reduce *P. aeruginosa* load ex vivo and that EDTA is highly active in suppressing the production of relevant bacterial virulence factors, suggesting a dual antibacterial and anti-virulence potential of the combination.

## 2. Results

### 2.1. Killing Activity of lin-SB056-1 in Combination with Ethylenediaminetetraacetic Acid (EDTA) against Endogenous P. aeruginosa

*P. aeruginosa* strains were isolated from the sputum of six PCD patients known to be chronically infected with the bacterium. All the strains displayed a mucoid phenotype and different antibiotic susceptibility profiles (Supplementary Table S1).

**Figure 1:**
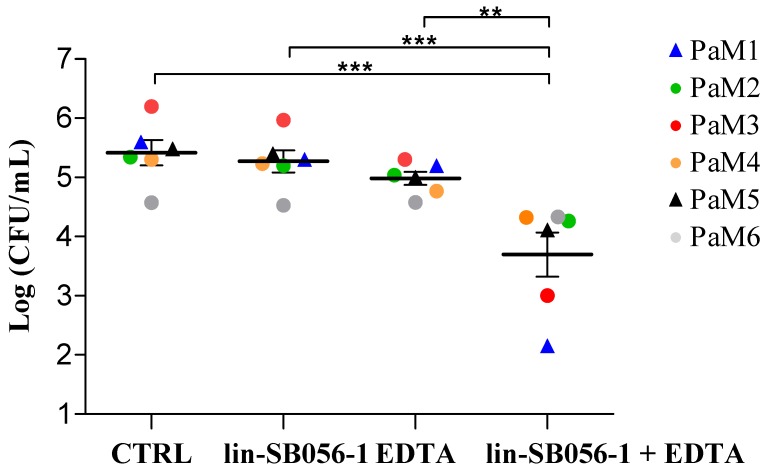
Antibacterial activity of peptide lin-SB056-1, ethylenediaminetetraacetic acid (EDTA), and both in combination against endogenous *P. aeruginosa* in primary ciliary dyskinesia (PCD) sputum. The effect of lin-SB056-1 and/or EDTA after 1.5 h of incubation in six diluted (1:5) sputum samples was assessed against endogenous *P. aeruginosa* strains (PaM1 to PaM6) by colony forming unit (CFU) counting. Lin-SB056-1 was tested at 25 μg/mL in combination with 0.625 mM EDTA against PaM1 and PaM5 strains (triangles), and with 1.25 mM EDTA against PaM2, PaM3, PaM4, and PaM6 strains (dots). Control (CTRL): bacteria incubated in diluted sputum only. Individual sputum samples are identified with different colors. Results represent the mean of 6 sputa done in duplicate. Error bars indicate the standard error of the mean. ** *p* < 0.01, *** *p* < 0.001 (one-way analysis of variance (ANOVA) followed by the Tukey–Kramer post-hoc test).

Diluted sputum (5-fold) from each patient was incubated for 1.5 h with the peptide at 25 μg/mL, alone or in combination with EDTA (0.625 or 1.25 mM), and the colony forming unit (CFU) number of *P. aeruginosa* surviving the treatment was detected. During the incubation time, endogenous *P. aeruginosa* did not grow in PCD sputum. While the peptide and EDTA were almost inactive when used alone, their combination exerted a significant synergistic killing effect against endogenous *P. aeruginosa*, although with different efficacy depending on the sputum sample (Figure 1). When compared to the corresponding controls, the reduction in CFU number caused by the combination ranged from approximately 0.3 Log-units (50% reduction, grey dot) to 3 Log-units (99.9% reduction, blue triangle) (Figure 1).

### 2.2. Effects of EDTA and lin-SB056-1 on Virulence Factors’ Production by P. aeruginosa PaM1 and PaM5

Preliminary experiments indicated that PaM1and PaM5 strains are able to produce high levels of most of the virulence factors analyzed; therefore, these strains were selected to evaluate the effect of sub-inhibitory concentrations of EDTA on virulence factors’ production. To this aim, we first evaluated the susceptibility of PaM1 and PaM5 strains to EDTA in liquid medium, in terms of minimum inhibitory concentration (MIC). A concentration of 1.25 mM EDTA was able to inhibit visible bacterial growth (MIC), while the concentrations of 0.075 and 0.15 mM were sub-inhibitory and therefore, were selected for the subsequent experiments.

**Figure 2:**
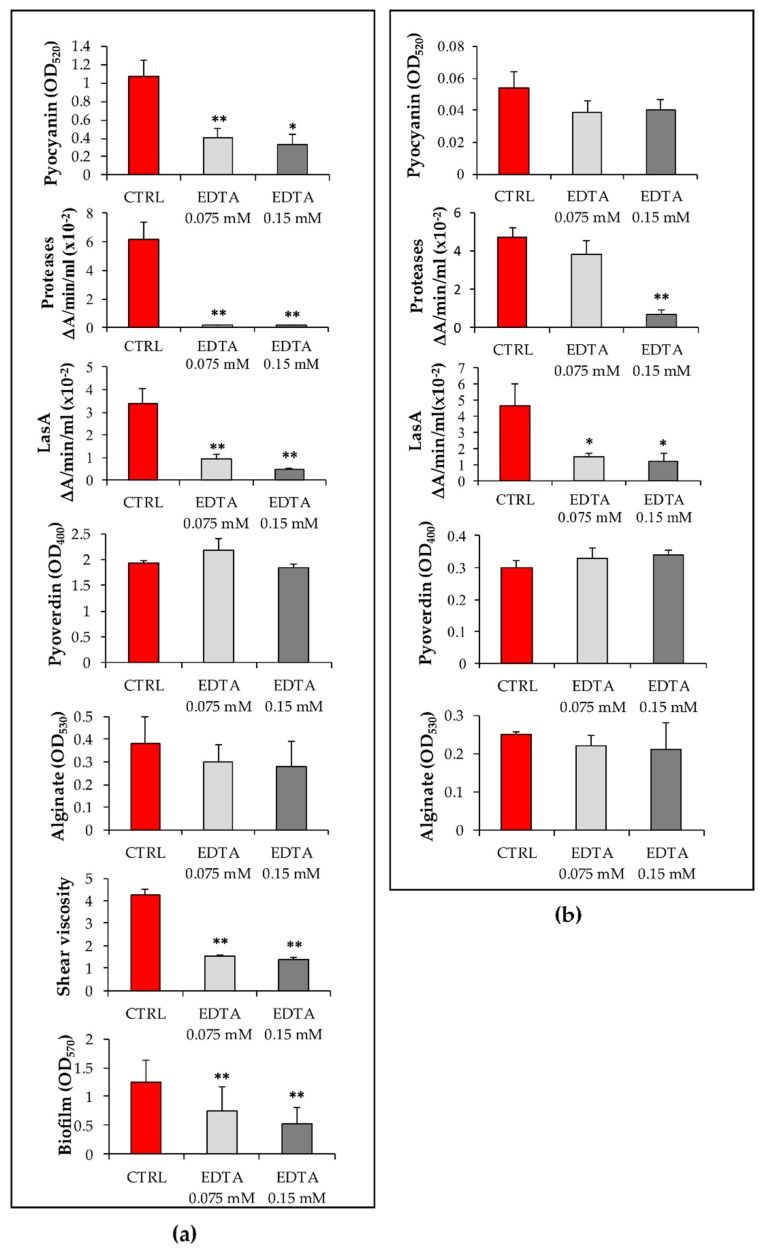
Effects of EDTA on virulence factor production by (**a**) PaM1 and (**b**) PaM5 strains. PaM1 and PaM5 cultures were incubated at 37 °C in the presence or absence of EDTA for 72 h. Following incubation, OD_600_ was measured prior the quantification of the virulence factors in culture supernatants (see the Materials and Methods Section for details). Values obtained were normalized by multiplying them by the ratio between OD_600_ of the control/OD_600_ of the corresponding EDTA-treated samples and reported as mean +/- SEM of three independent experiments. CTRL: bacteria incubated without EDTA; * *p* < 0.05, ** *p* < 0.01 (one-way ANOVA followed by the Tukey–Kramer post-hoc test).

Pyocyanin is a greenish pigment secreted by *P. aeruginosa* that enhances the inflammatory response and causes tissue damage in the host [21]. As shown in Figure 2a, EDTA at the concentrations of 0.075 and 0.15 mM, highly inhibited pyocyanin production by the PaM1 strain at 72 h (by 62% and 70%, respectively) as compared to the untreated cells. Regarding the PaM5 strain, which was a low pyocyanin producer (Figure 2b), EDTA at both concentrations caused a reduction of approximately 40% in the production of such pigment, although the difference did not reach statistical significance compared to the untreated cells.

*P. aeruginosa* produces and secretes a number of proteases, such as LasA, elastase B (LasB), protease IV, and alkaline protease, which are considered important virulence factors as they damage host tissues and interfere with host antibacterial defense mechanisms [22]. The total proteolytic activity of PaM1 was completely abolished in the presence of either 0.075 or 0.15 mM EDTA (Figure 2a). Similarly, EDTA significantly reduced the proteolytic activity of the PaM5 strain but only at the concentration of 0.15 mM (Figure 2b).

LasA is a zinc-dependent metalloprotease secreted by *P. aeruginosa*. It exhibits a staphylolytic activity, enhances the elastolytic activity of LasB in vivo, and induces shedding of syndecans, a family of cell surface heparan sulfate proteoglycans, from host cell surfaces [23]. LasA activity of both the PaM1 and PaM5 strains was significantly inhibited in the presence of 0.075 and 0.15 mM EDTA, with a reduction of approximately 70% and 80%, as compared to the untreated control, respectively (Figure 2a,b). Further experiments were undertaken in order to evaluate whether the reduction of LasA activity was ascribable to the inhibition of protease synthesis or, rather, to the inhibition of the enzyme activity due to zinc chelation by EDTA (Figure S1). To this aim, the assessment of the PaM1 staphylolytic activity was performed in the presence of exogenously added zinc (0.1 mM ZnSO_4_). When ZnSO_4_ was added directly in the enzyme assay, at the end of the incubation period (72 h), no significant increase in LasA activity was observed, suggesting that the low levels of LasA activity detected in the presence of EDTA were likely due to inhibition of protein synthesis and not to the chelation of the enzyme cofactor. In contrast, when ZnSO_4_ was added at the beginning of the incubation of PaM1 with EDTA, LasA activity was restored (Supplementary Figure S1), indicating that the excess of zinc could overcome the inhibitory effect of EDTA. Overall, these data suggest that EDTA may act by interfering with the expression/procession of LasA protease by PaM1 strain rather than by sequestering the zinc cofactor.

Pyoverdin is a chelator involved in iron binding and cellular uptake in a low-iron environment [24]. EDTA at both concentrations tested did not reduce the level of pyoverdin in culture supernatants of both *P. aeruginosa* strains (Figure 2a,b).

Studies on mucoid *P. aeruginosa* isolates have shown that alginate plays a critical role in biofilm establishment and persistence by protecting bacteria against antibiotics and phagocytosis [25,26]. Although EDTA did not inhibit the production of alginate in culture supernatants of both strains (Figure 2a,b), it was able to significantly reduce the viscosity of PaM1 culture supernatants at both concentrations tested (Figure 2a).

Finally, EDTA was tested for its antibiofilm activity against the PaM1 strain. A reduction of 40% and 57% in PaM1 biofilm formation was observed in the presence of 0.075 and 0.15 mM EDTA, respectively (Figure 2a).

The impact of lin-SB056-1 on the production of virulence factors by PaM1 and PaM5 strains was also evaluated. As reported in Table S2, the peptide at sub-inhibiting concentrations did not reduce the production of any of the virulence factors analyzed for both bacterial strains tested.

## 3. Discussion

Similar to CF patients, eradication of chronic *P. aeruginosa* infection in PCD lungs is hardly obtained, and the reduction of bacterial density during chronic colonization or exacerbations is often the aim of the antimicrobial therapy [3]. In previous studies, we have shown that the combination of lin-SB056-1/EDTA possesses antimicrobial activity against *P. aeruginosa* in artificial sputum medium and prevents *P. aeruginosa* biofilm formation in an in vivo-like three-dimensional (3D) lung epithelial cell model [16,27]. Despite resembling the airway mucus, artificial sputum media normally behave like Newtonian fluids lacking many of the intramolecular interactions and covalent cross-links that give respiratory secretions their viscoelastic characteristic [28]. Furthermore, the genotype and physiological state of *P. aeruginosa* cells found in vivo may significantly differ from those of bacteria grown in laboratory media [29]. Hence, in this study, we sought to validate the anti-pseudomonal activity of the lin-SB056-1/EDTA combination in conditions more closely resembling the environment found in vivo. To this aim efficacy of the combination was tested ex vivo, against endogenous *P. aeruginosa* in the sputum of PCD patients. Differently from artificial sputum medium, patients’ sputum contains host/bacterial components such as cell-derived factors, normal flora, inflammatory mediators, proteases, or peptidases that may exert an additional inhibitory effect on the peptide’s activity. Nevertheless, herein, we showed that the combination of lin-SB056-1 and EDTA at sub-active and non-cytotoxic concentrations [16,27] determined a significant reduction in *P. aeruginosa* load in sputa of PCD chronically infected patients, despite certain differences in the level of reduction among different sputum samples being observed. Such differences are not surprising considering that inter-patient variables (e.g., clinical stage and severity of lung infection, bacterial load, sputum sample composition, and consistency) were not standardized in our experiments, in the attempt to mimic conditions found during the actual antimicrobial therapy. The mechanisms of the synergistic effect of EDTA on the peptide’s activity might be multiple. Due to the chelation of divalent cations from their binding sites in lipopolysaccharide (LPS), EDTA may destabilize the bacterial outer membrane, thus increasing the permeability to lin-SB056-1 molecules and their interaction with the bacterial membranes. In addition, at least part of the synergistic effect observed in sputum could be ascribed to the ability of EDTA to reduce sputum viscosity, thus favoring peptide diffusion [16]. Finally, EDTA could also neutralize the inhibitory effect of sputum on the peptide’s activity, sequestering cations that may interfere with the electrostatic interactions of the peptide with bacterial surface [9,10]. The possible use of EDTA in the treatment of pulmonary infections and its safety as an adjuvant has been highlighted in previous in vivo studies [30,31]. For instance, Liu and coworkers demonstrated, in the guinea pig model, that EDTA (30 mg/kg intraperitoneally injected) plus ciprofloxacin (4 µg/mL administered by inhalation) significantly reduced the *P. aeruginosa* CFU number per gram of lung tissue as compared to the single treatment groups [31].

Interestingly, the results obtained in this study clearly demonstrated that EDTA could not only favor the activity of lin-SB056-1 in ex vivo conditions, but could also reduce in vitro, at sub-inhibitory concentrations, the production of several *P. aeruginosa* virulence factors (i.e., pyocyanin, total protease and LasA), which are known to play a crucial role in the pathogenesis of *P. aeruginosa* infections. The action of EDTA as an anti-virulence molecule could be ascribed to its capacity of binding divalent cations, many of which are critical for the expression/processing of virulence factors of *P. aeruginosa*. In particular, the reduction of pyocyanin observed in the presence of EDTA is in line with previous reports demonstrating a positive correlation between calcium levels and the expression of proteins involved in the pathway of pyocyanin biosynthesis [32]. Analogously, the reduction of proteases by EDTA is in agreement with previous observations reporting that zinc ions are important for the efficient production and processing of different proteases, such as LasA, LasB, and protease IV [19,33].

On the contrary, EDTA did not inhibit pyoverdin production, in agreement with the observation that neither calcium nor magnesium enhances pyoverdin production [34,35]. Interestingly, although EDTA did not inhibit alginate production, it was able to markedly reduce the viscosity of culture supernatants of the PaM1 strain. It can be hypothesized that this effect may be due to the sequestration of calcium ions that are crucial for alginate cross-linking [36]. A similar mechanism may be involved in the ability of EDTA to reduce the formation of PaM1 biofilm, confirming previous observations in which EDTA significantly reduced biofilm formation by a mucoid strain of *P. aeruginosa* either in vitro or in a guinea-pig model of lung infection [31]. Overall, the ability of EDTA to reduce the accumulation and/or activity of important virulence factors might contribute to limit the pathogenicity of *P. aeruginosa*.

In conclusion, in the present study, we demonstrated that the lin-SB056-1/EDTA combination is able to significantly reduce *P. aeruginosa* load in PCD sputum, and that EDTA decreases the production of relevant virulence factors of mucoid *P. aeruginosa* in vitro. Such results suggest a dual antimicrobial and anti-virulence effect of the lin-SB056-1/EDTA combination and highlight the possible use of EDTA as an adjuvant in the treatment of chronic *P. aeruginosa* lung infections.

## 4. Materials and Methods

### 4.1. Sputum Collection and Treatment

The sputum samples were collected by spontaneous expectoration from six PCD patients following informed consent. The study was conducted in accordance with the Declaration of Helsinki, and the protocol was approved by the Ethics Committee of Pisa (Protocol number 62532, 11.06.2016). PCD patients included in this study (median age: 34 years) were chronically infected by *P. aeruginosa* and characterized by frequent relapses of infection. A volume of 0.5–1 mL of sputum was collected from each patient after interruption (at least 14 days) of the antibiotic therapy regimen. For easier handling of the samples, the dense and sticky sputa were diluted five-fold in sodium phosphate buffer 10 mM, pH 7.4 (SPB). Samples were plated on cetrimide (Sigma Aldrich, Saint Louis, MO, USA) and MacConkey (Oxoid Basingstoke, Hampshire, UK agar to confirm the presence of *P. aeruginosa* and assess the mucoid phenotype of the colonies, respectively.

### 4.2. Peptide and EDTA Solutions

Lin-SB056-1 peptide (KWKIRVRLSA-NH_2_) was purchased from Peptide Protein Research, Ltd. (Fareham, UK) with a purity of 98%. EDTA (disodium salt) was obtained from Sigma-Aldrich. A stock solution of disodium-EDTA (0.5 M) was prepared in milli-Q water by adjusting the pH to 8.0 with NaOH (Sigma-Aldrich). The stock solution was then diluted in milli-Q water to obtain a working solution of 50 mM that was sterilized and stored at 4 °C.

### 4.3. Susceptibility Testing

Identification and susceptibility testing of *P. aeruginosa* strains isolated from sputum samples (PaM1, PaM2, PaM3, PaM4, PAM5, PaM6) were performed by MALDI-TOF (Bruker Daltonics, Bremen, Germany) and VITEK 2 automatic instruments (BioMerieux, Lyon, France), respectively (Table S1). Determination of minimum inhibitory concentration (MIC) of EDTA towards PaM1 and PaM5 strains was performed according to the standard microdilution method in Muller–Hinton broth (Oxoid) [37]. The MIC was defined as the lowest concentration of EDTA that completely inhibited visible growth of bacteria after 24 h of incubation.

### 4.4. Bactericidal Assay in Patients’ Sputum

An aliquot of each PCD sputum was serially diluted and plated on selective cetrimide agar, to assess the CFU number of endogenous *P. aeruginosa* at time 0. After that, a volume of 90 µL of diluted (1:5) sputum of each patient was incubated with sub-bactericidal concentrations of peptide and EDTA, used alone or in combination, for 1.5 h at 37 °C. Following incubation, samples were serially diluted and plated on selective cetrimide agar for assessing the *P. aeruginosa* CFU number.

### 4.5. Assays for Evaluation of Virulence Factors in Culture Supernatants

Colonies of mucoid strains PaM1 and PaM5 grown on MacConkey agar were suspended in Luria Bertani (LB) broth (Sigma-Aldrich) to obtain an OD_600_ of 0.1. Cultures were incubated in the presence or in the absence of EDTA (0.075 or 0.15 mM) in static conditions at 37 °C for 72 h. Following incubation, the OD_600_ of the cultures was determined to account for bacterial density. After that, cultures were centrifuged at 10,000× *g* for 20 min at room temperature and culture supernatants were used for the quantification of virulence factors. The same protocol was followed to evaluate the eventual effects of linSB056-1 on the production of virulence factors. To this aim, the peptide was added to PaM1 and PaM5 cultures at the concentration of 6.25 µg/mL and 12.5 µg/mL that were sub-inhibitory for both bacterial strains.

Pyocyanin was extracted from cell-free supernatants with subsequent exposure to chloroform and 0.2 N hydrochloric acid (Sigma-Aldrich) and quantified at OD_520_ nm, as previously described [38].

Total proteolytic activity was determined using a modified skim milk assay [39]. Briefly, culture supernatants of PaM1 and PaM5 strains (0.5 mL) were incubated with 0.5 mL skim milk (Sigma-Aldrich) (1.25%) at 37 °C for 30 min and turbidity was measured at OD_600_ nm. The decrement in turbidity due to proteolytic activity was expressed as ΔA/min/mL.

Secreted LasA of *P. aeruginosa* has a staphylolytic activity, i.e., it causes a decrement in the OD_600_ of a culture of *Staphylococcus aureus*. LasA activity was assessed by evaluating the ability of cell-free supernatants from *P. aeruginosa* exposed or not exposed to EDTA to lyse boiled cells (intact) of *S. aureus* American Type Culture Collection (ATCC) 33591 and expressed as ΔA/min/mL [40]. Due to the role of zinc as a cofactor of LasA, in some experiments, the staphylolytic activity in the presence of EDTA was evaluated by adding 0.1 mM ZnSO_4_ (Sigma-Aldrich) to the enzyme assay.

Pyoverdin was quantified by measuring the OD_400_ of cell-free supernatants [41].

The quantification of alginate was performed by carbazole-borate assay according to Heidari et al. [42]. Shear viscosity of culture supernatants was assessed by rheometric measurement at 25 °C, applying a shear stress of 1 Pa/s on 150 µl of supernatant using a gap between the rheometer plates of 52 µm (Rheometer Scientific RM500, Reologica Instruments AB, Lund, Sweden).

The value obtained for each virulence factor was multiplied by the ratio OD_600_ of the control/OD_600_ of the sample, to normalize for small differences in the culture densities between the controls and the EDTA-exposed samples after 72 h of incubation.

### 4.6. Biofilm Inhibition Assay

*P. aeruginosa* PaM1 grown in tryptone soy broth (TSB) for 48 h at 37 °C was diluted 1:20 in TSB supplemented with 0.25 mM CaCl_2_. Bacterial suspensions were inoculated into flat-bottom polystyrene 96-well microplates (Corning Costar, Lowell, MA, USA) in the absence (negative control) or in the presence of EDTA at sub-inhibiting concentrations (0.075 and 0.15 mM). Microplates were incubated statically at 37 °C for 48 h and biofilm biomass was estimated by crystal violet (CV) (Sigma-Aldrich) staining assay, as previously described [43].

### 4.7. Statistical Analysis

Data reported in Figure 1 represent the mean of 6 experiments done in duplicate. Figure 2 and Supplementary Figure S1 depict the data obtained from three independent experiments, while Table S2 reports the mean of two independent experiments. Differences between mean values of groups were evaluated by one-way analysis of variance (ANOVA) followed by the Tukey–Kramer post-hoc test. A *p*-value < 0.05 was considered statistically significant. Data analysis was performed with GraphPad In Stat (GraphPad Software, La Jolla, CA, USA).

## Supplementary Materials

Supplementary Materials can be found at https://www.mdpi.com/1422-0067/21/1/69/s1. Table S1: Patients’ information, colony phenotype, and resistance profile of *P. aeruginosa* strains isolated from PDC sputum; Table S2: Effects of lin-SB056-1 on the production of virulence factors by PaM1 and PaM5 strains; Figure S1: Effect of exogenously added Zinc on LasA staphylolytic activity of PaM1 strain in the presence of EDTA.

## Abbreviations

ATCC	American Type Culture Collection
AMPs	Antimicrobial peptides
ANOVA	Analysis of variance
CF	Cystic fibrosis
CFU	Colony forming units
CV	Crystal violet
EDTA	Ethylenediaminetetraacetic acid
LB	Luria-Bertani broth
LPS	Lipopolysaccharide
MIC	Minimum Inhibitory Concentration
OD	Optical density
PCD	Primary Ciliary Dyskinesia
SPB	Sodium Phosphate Buffer
TSB	Tryptone Soy Broth
