# Supplementary material for: Targeting Pseudomonas aeruginosa in the Sputum of Primary Ciliary Dyskinesia Patients with a Combinatorial Strategy Having Antibacterial and Anti-Virulence Potential
Source: Int J Mol Sci. 2019 Dec 20;21(1):69. doi: 10.3390/ijms21010069 (PMC6981532; doi:10.3390/ijms21010069)
Supplement: Supplementary file 1 [file ijms-21-00069-s001.zip › ijms-671161-SI.pdf]

Supplementary materials

Table S1: Patients' information, colony phenotype, and resistance profile of *P. aeruginosa* strains isolated from PDC sputum

Patient (sex)	Age at the diagnosis	Age at the time of sampling	*P. aeruginosa* strain isolated from sputum	Colony phenotype	Resistance profile
1 (F)	21y;2mo	23y;1mo	PaM1	mucoid	none
2 (F)	27y;8mo	34y;6mo	PaM2	mucoid	FOS-LVX-TOB
3 (M)	9y;7mo	16y;6mo	PaM3	mucoid	CIP-FOS-IMP-TZP
4 (M)	32y;2mo	33y;1mo	PaM4	mucoid	AMK-CFP-ERT-GEN-PIP-TZP-TOB-AZT
5 (M)	42y;2mo	47y;10mo	PaM5	mucoid	FEP-CAZ-CIP-FOS-GEN-IMP-LVX-MER-TZP
6 (F)	28y;3mo	29y;7mo	PaM6	mucoid	LVX

AMK: amikacin; AZT: azithromycin; CAZ: ceftazidime; CIP: ciprofloxacin; CFP: cephapirin; ERT: ertapenem;FEP: cefepime; FOS: fosfomycin;GEN: gentamycin; IMP: imipenem; LVX: levofloxacin; MER:meropenem;PIP: piperacillin; TOB: tobramycin;;LVX: levofloxacin; TOB: tobramycin; TZP: piperacillin/tazobactam. y:years; mo:months

Table S2: Effects of lin-SB056-1 on the production of virulence factors by PaM1 and PaM5 strains. The data represent the mean of two independent experiments.

	**PaM1**			**PaM5**		
	**Lin-SB056-1**			**Lin-SB056-1**		
	**0**	**6.25 µg/ml**	**12.5 µg/ml**	**0**	**6.25 µg/ml**	**12.5 µg/ml**
**Pioverdin**[a]	0.640	0.570	0.510	0.260	0.250	0.220
**Pyocianin**[b]	0.490	0.320	0.300	0.038	0.031	0.040
**LasA**[c]	0.040	0.040	0.040	0.032	0.045	0.040
**Proteases**[d]	0.056	0.052	0.050	0.032	0.032	0.030
**Alginate**[e]	0.770	0.710	0.840	0.170	0.110	0.120

[a] OD_{400} of cell-free supernatants after 72 h of incubation with different concentrations of the peptide; [b]OD_{520} after proper treatment of the supernatant; [c]Decrement of absorbance at 600 nm of a suspension of *S. aureus* cells (ΔA/min/ml);

[d]Decrement of absorbance at 600 nm of a skin milk solution (ΔA/min/ml); [e]OD_{530} after proper treatment of the supernatant. See Materials and Methods for details

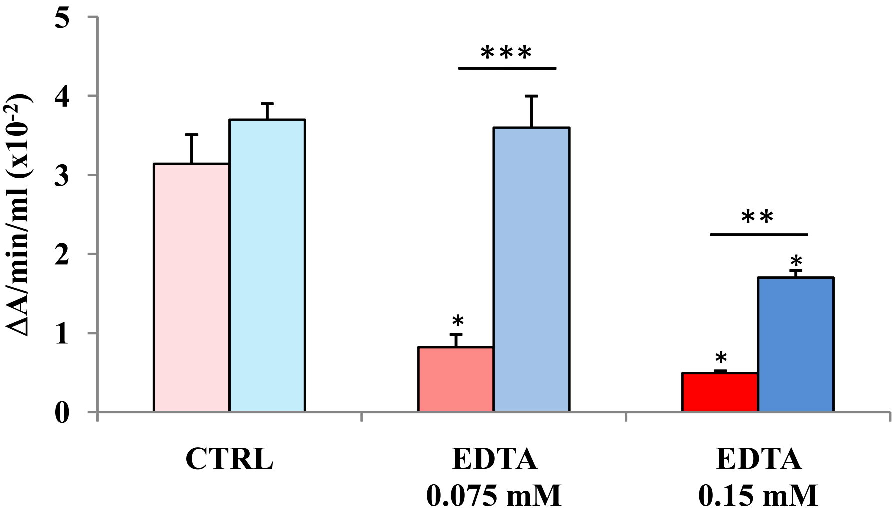

Figure S1. Effect of exogenously added Zinc on LasA staphylolytic activity of PaM1 strain in the presence of EDTA. $ZnSO_4$ (0.1 mM) was added in the enzyme assay at the end of the incubation period (72 h) (red bars) or in the bacterial culture at the beginning of the incubation period (blue bars). LasA activity was assessed by evaluating the ability of cell-free supernatants to lyse boiled cells (intact) of *Staphylococcus aureus* ATCC 33591. The decrement of OD_{600} due to cell lysis was expressed as ΔA/min/ml. Data obtained were normalized by multiplying them by the ratio between OD_{600} of the control control/OD_{600} of the corresponding EDTA-treated samples, and reported as mean +/- SEM of three independent experiments. CTRL: bacteria incubated without EDTA; *$p<0.05$, **$p<0.01$, ***$p<0.001$ (one-way ANOVA followed by Tukey-Kramer post-hoc test).
